# Automatic needle detection using improved random sample consensus in CT image‐guided lung interstitial brachytherapy

**DOI:** 10.1002/acm2.13231

**Published:** 2021-03-25

**Authors:** Yongnan Zheng, Shan Jiang, Zhiyong Yang, Lin Wei

**Affiliations:** ^1^ School of Mechanical Engineering Tianjin University Tianjin China

**Keywords:** lung brachytherapy, computed tomography, improved random sample consensus, needle localization

## Abstract

**Purpose:**

To develop a method for automatically detecting needles from CT images, which can be used in image‐guided lung interstitial brachytherapy to assist needle placement assessment and dose distribution optimization.

**Material and Methods:**

Based on the preview model parameters evaluation, local optimization combining local random sample consensus, and principal component analysis, the needle shaft was detected quickly, accurately, and robustly through the modified random sample consensus algorithm. By tracing intensities along the axis, the needle tip was determined. Furthermore, multineedles in a single slice were segmented at once using successive inliers deletion.

**Results:**

The simulation data show that the segmentation efficiency is much higher than the original random sample consensus and yet maintains a stable submillimeter accuracy. Experiments with physical phantom demonstrate that the segmentation accuracy of described algorithm depends on the needle insertion depth into the CT image. Application to permanent lung brachytherapy image is also validated, where manual segmentation is the counterparts of the estimated needle shape.

**Conclusions:**

From the results, the mean errors in determining needle orientation and endpoint are regulated within 2° and 1 mm, respectively. The average segmentation time is 0.238 s per needle.

## 1 INTRODUCTION

Lung cancer has been the most common incident cancer with over 4 million new cases diagnosed and 2 million cancer deaths taking place in China each year.^1^ As an important branch of radiotherapy, interstitial brachytherapy has been widely used for cancer therapeutic, such as lung, liver, prostate, and many other organs with great conformality, short treatment time, and high rate of local dose control.^2^, ^3^, ^4^

The standard workflow of the lung interstitial brachytherapy generally consists of three parts: First, an optimal dose treatment plan should be made by the physicist based on the treatment planning system.^5^ Second, all the needles are inserted into the target along the directional slots on the coplanar template. The radioactive sources are implanted into planned positions sequentially through the hollow shaft of the needle. Finally, verifying the quality of the operation by postoperative CT.^6^

**Fig. 1:**
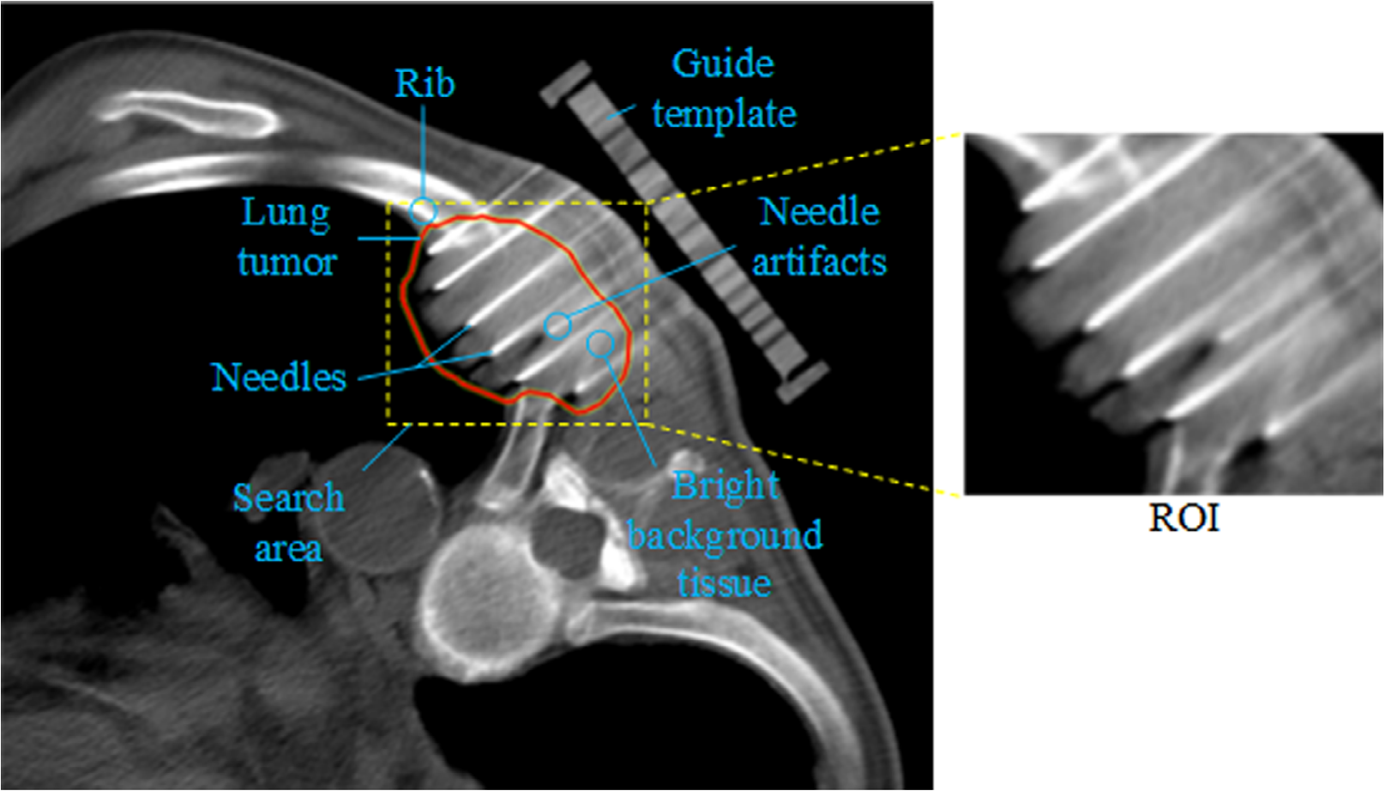
An instance of the needle appearance during lung interstitial brachytherapy performed on CT images. The ROI is displayed on the right.

Placing all seeds in the planned positions to ensure sufficient dose to cover the target, while maintaining a low dose of the organs at risk is the key to surgery success.^7^ However, preoperative plan cannot be implemented well due to factors such as tissue deformation and needle deviation.^8^ Suboptimal dose distribution leads to decreased biochemical control and high risk of damage to surrounding tissues. An image‐based automatic needle detection algorithm makes it possible to perform efficient intraoperative dose correction based on the actual needle paths. In the treatment planning system, the planned seeds can be imported into the corresponding detected needle paths for dose calculation. If any dose deficiency and hot spot are identified within the lung target, position adjustment of seeds and additional needles will be done. However, needle artifacts, image attenuation, signal dropouts, and low contrast between the needle and the ribs have brought many obstacles to automatic needle detection in CT data, as shown in Fig. 1. Therefore, accurately and quickly identifying the needle from a large amount complex of CT data has become the nodus of this technique.

A wide variety of research studies have been performed on automatic long straight surgical instruments segmentation from two‐dimensional (2D) and three‐dimensional (3D) ultrasound (US) images. Radon, Hough transform (HT), and parallel projections are three common detection algorithms. An example that using a form of the Radon transform, Barva et al.^9^ proposed that the electrode axis can be found through maximizing parallel integral projection (PIP). Although the accuracy of this method is high with tip error within 0.2‐0.3 mm, it is not a pragmatic approach in a clinical environment due to a long processing time in MATLAB (tens of minutes). Additional PIP speedup achieved through harnessing the computational power of the GPU and using a fast search schema to track the surgical instruments real time.^10^ Uhercik et al.^11^ presented a multiresolution PIP method, achieving faster electrode axis localization in 3D US data than generalized PIP and yet maintain the same accuracy. Qiu et al.^12^ used the 3D HT‐based method for locating the prostate therapy needle, which achieved endpoint localization accuracy of 1 mm in *vivo* patient images and the mean time cost is 2 s. By modifying the HT algorithm with coarse‐fine search strategy and a four parameter representation of lines in 3D space, Zhou et al.^13^ segmented needles quickly, accurately, and robustly. Quick randomized Hough transform (RHT) reduced the computational effort to 1 s in a 35 MB data by doing the RHT only on coarse resolution volumes.^14^ Okazawa et al.^15^ generalized the HT to address needle curvature in 2D ultrasound images. Ding et al.^16^ used two orthogonal image projections to segment needles from 3D US images and a similar segment algorithm can be used to detect curved needle found by Aboofazeli et al.^17^ For higher preciseness, these methods of projection need images with low noise and good contrast. Regarding localizing the needle position and estimate the endpoint in real time, Yan et al.^18^ presented the shape information and level set technique, which had been validated in prostate phantoms.

The general disadvantages of aforementioned methods are their high sensitivity to interference, computational complexity, and focusing on single needle separation. Hence, a more robust and computational efficient approach that functions well within a complicated environment is still required. In this paper, a modified needle detection algorithm has been proposed and evaluated, which estimates the entire needle shape utilizing image preprocessing and an improved random sample consensus (RANSAC) algorithm. RANSAC has been successfully used in surgical tool localization from 3D US images^19^ and multiple transverse US images,^20^ but so far has not been applied to CT transverse data. In this algorithm, the iteration process has been accelerated through pretesting technology to improve the overall needle segmentation efficiency. Meanwhile, the accuracy is guaranteed by combining locally optimized RANSAC^21^ with principal component analysis (PCA).^22^ Furthermore, by cycling out of the current best model inliers, all needles in a single transverse image are detected. Finally, the algorithm is comprehensively evaluated in simulation data, physical phantom, and in *vivo* CT‐guided lung brachytherapy images.

## 2 MATERIALS AND METHODS

### 2.1 CT image preprocessing

The distance between adjacent needles is usually 5 or 10 mm, which has been proved with minimal damage to the human body.^5^ The thickness of CT slices used for lung brachytherapy is normally 5 mm to ensure that all needles and seeds can be detected while obtaining distinct tissue imaging. Using a locating device and lasers to adjust and position the coplanar template before scanning can ensure that each needle is exactly on the CT slice. It is assumed that the acquired images contain all the needles, and the count of needles on each slice is known. The planned contours are manually adjusted by the physicist to account for the anatomic deformation, and then imported into intraoperative CT images. According to literature,^23^ we found that the deformation of the 18‐gauge brachyneedle occurs mainly in the direction perpendicular to the bevel of the needle and no bending occurs during insertion.

#### 2.1.1 Region of interest (ROI) extraction

Comparing with the manual extraction, a semiautomatic ROI detection method is used based on the modified target contour. First, a smallest enclosing rectangle of the target area is calculated through the pixels on the contour. Then, the rectangle is expanded by 3 mm to ensure all the needle information within the ROI. If any needle point candidates are located outside the enlarged rectangle, the expansion distance will be increased manually. In the end, the image surrounded by the updated rectangle is cropped for subsequent processing. A ROI for one of the clinical CT images is shown in Fig. 1.

#### 2.1.2 Contrast enhancement

**Fig. 2:**
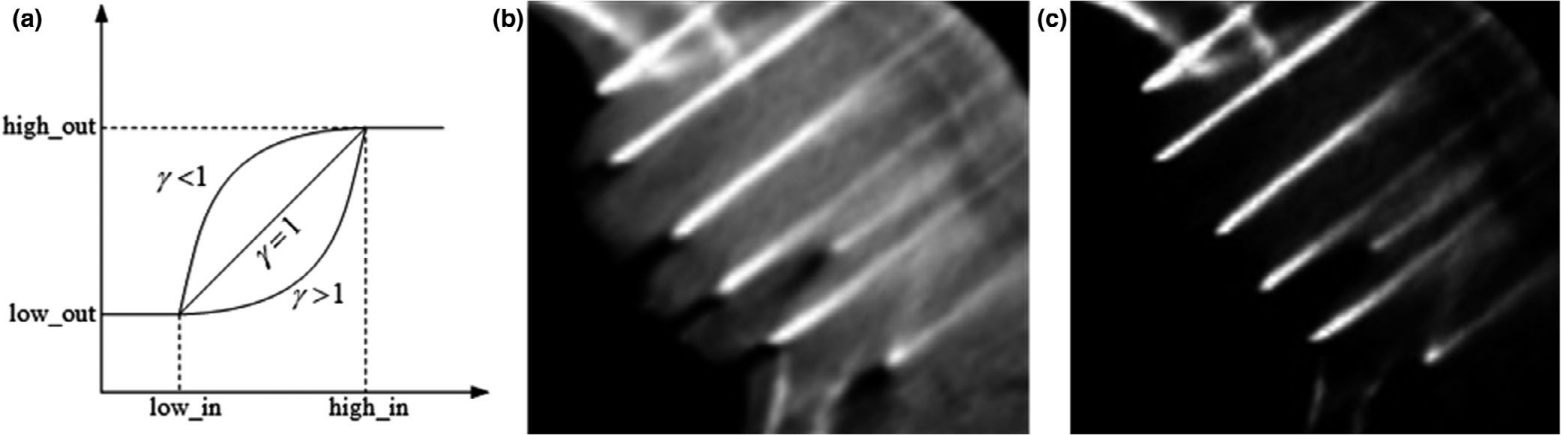
(a) is the principle of the contrast mapping function, (b) is the original cropped image, and (c) refer to the contrast enhanced image.

In order to improve the contrast between the needle and the exterior background noise, an intensity mapping function is applied to the cropped image after localizing the search area. The function is

(1) $$i_{f}=\left(i_{high\_out}‐i_{low\_out}\right){\left(\frac{i‐i_{low\_in}}{i_{high\_in}‐i_{low\_in}}\right)}^{\gamma }+i_{low\_out}$$

with $i_{f}$, i, $i_{low\_in}$, $i_{high\_in}$, $i_{low\_out}$, $i_{high\_out}$ representing the normalized intensity value in the range $\left[0,1\right]$. $i_{f}$ refers to the pixel intensities desired in the transformed image and i represents the original pixel intensities. The range $\left[i_{low\_in},i_{high\_in}\right]$ specifies the minimum and maximum saturation thresholds in the input intensities. The value $i_{low\_out}$ and $i_{high\_out}$ defines the intensities span of the desired spectrum. The significance of the transformation is shown in Fig. 2a. It maps the intensity values in inchoative grayscale image to new values in the transformed ROI. In the transformation process, the original pixels with intensities smaller than $i_{low\_in}$ are given the value of intensity $i_{low\_out}$. Likewise, pixels having the intensities higher than $i_{high\_in}$ are assigned to $i_{high\_out}$. As for the parameter γ, which defines the shape of the convert curve. When γ<1, the pixel intensities of the low‐intensity are expanded and compressing those of the high‐intensity pixels. Contrarily, when γ>1, the low‐intensity pixels are compressed and the high‐intensity pixels are expanded.

In our algorithm, the values $i_{low\_in}$ and $i_{high\_in}$ are chosen to 15% and 100% of the maximum intensity value 1, and the desired spectrum is set to range $\left[0,1\right]$. By setting γ>1, additional expansion toward higher intensity pixels, allowing for enhancing contrast between the needle and external background noise. Fig. 2b,c present the comparison between the original cropped image and the contrast enhanced image.

#### 2.1.3 Thresholding process

After process of contrast enhancement, the histogram of the gray distribution in the ROI is obtained. Based on thresholding α, intensities $I\left(x\right)\in \left[0,1\right]$ is separated into two disjoint sets: $X_{n}$ (needle pixels) and $X_{b}$ (background pixels).

(2) $$\left\{\begin{array}{c} X_{n}=\left\{x\in X:I(x)\geq \alpha \right\}\\ X_{b}=X‐X_{n} \end{array}\right.$$

where X is a series of pixel coordinate. Threshold α is identified based on empirical results, because the needle grayscale distribution of each patient case is different. Through Eq. (2), pixels with intensity larger than α are identified as candidate needle points. Note that $X_{n}$ just provides an approximate estimation of the candidate needle points and contains some false points. A modified RANSAC method for needle axis identification is described in the following section.

### 2.2 Needle axis segmentation

#### 2.2.1 Improved RANSAC algorithm for needle detection

Assuming that *N* interrelated data points are contained in a dataset *X*. RANSAC algorithm^24^ randomly samples a subset of size *m* from *X*, among them *m* is the minimum data collection for determining the parameterized model and its value is respond to the complexity of the geometry model. Once a model is presumed by calculation from the minimum dataset, entire data points in dataset *X* are used to examine the model and then determine the inliers of it. The sampling process will be repeated until meeting the final evaluation criteria. It means that the minimum samples required for a smallest subset without outliers can be screened at least on the given confidence probability *P*. Finally, the model with the highest support rate (most inliers) is the desired model. Supposing that the incorrect outlier occupancy rate is ε, and the probability of choosing an un‐contaminated m inliers sample point is ${\left(1‐\varepsilon \right)}^{m}$. Similarly, *k* samples are chosen and the probability of contamination of these sample data (at least one outlier) is ${\left(1‐{\left(1‐\varepsilon \right)}^{m}\right)}^{k}$. Therefore, to make sure that the probability of confidence is above *P*, the least number of samples *k* must satisfy the following relationship:

(3) $$k\geq \frac{\log(1‐P)}{\log(1‐{\left(1‐\varepsilon \right)}^{m})}$$

Through aforementioned step, the time complexity of RANSAC algorithm is considered to *T*:

(4) $$T=k*(T_{X}+T_{C}*N)$$

where $T_{X}$ is the time of each random sampling, $T_{C}$ is the average time of validating the model for each data point; N is total number of data points in X. In general, $T_{X}$ and $T_{C}$ are considered invariant for a specific issue, so the time complexity of RANSAC algorithm is decided by k and N.

From Eq. (3), we can see that k is exponential with ε and m. Under a certain confidence probability, the value of k and N will heighten with the increasing complexity of the data set and the model. Meanwhile, the time intricacy T will be increased accordingly. Hence, the technology of combination of preview model parameters evaluation and local optimization is proposed to improve the speed of the algorithm with a high accuracy.

#### 2.2.2 Preview model parameters evaluation technology

**Fig. 3:**
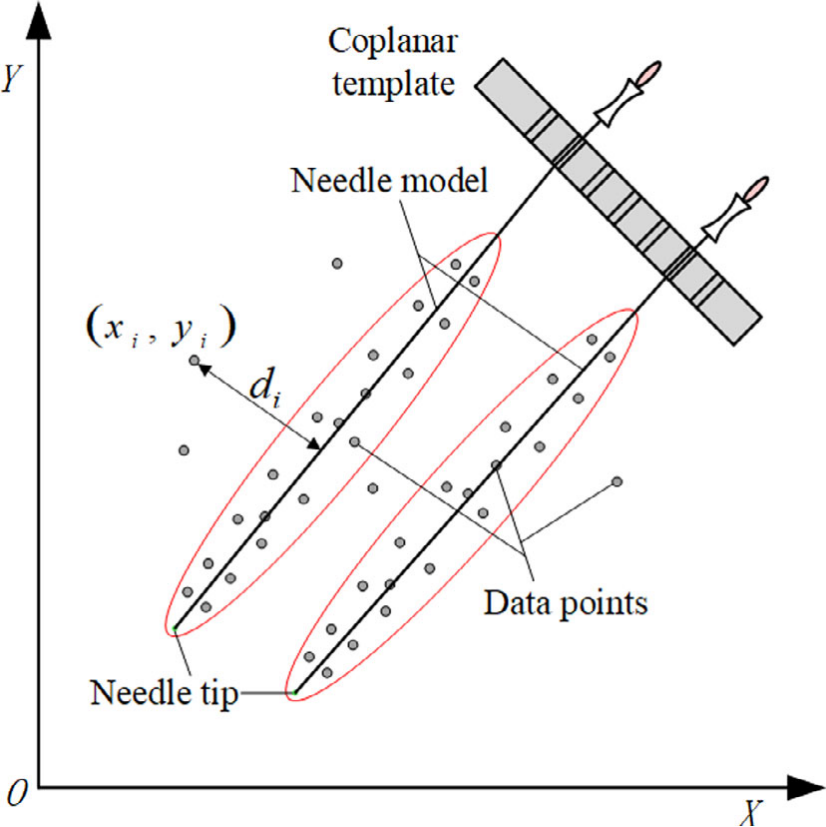
Diagram illustrating needle and $d_{i}$ for the ith point in the dataset X.

Assuming that the sample data for pretesting is n and the probability of existence at least $n_{f}$ inliers is $P_{t}$. In other words, at the confidence probability $P_{t}$, the inliers are at least $n_{f}$ for correct model under n tests. If the number of inliers is less than $n_{f}$, this model's parameters are contaminated by outliers, which no need to participate in the subsequent verification of all the data. For the correct model parameters, the probability of passing the pretesting is:

(5) $$P_{t}=1‐\sum_{i=0}^{n_{f}‐1}C_{n}^{i}\varepsilon^{\left(n‐i\right)}{\left(1‐\varepsilon \right)}^{i}$$

where $C_{n}^{i}$ is a combination of *i* data selected from sample data *n*. In the actual calculation, the maximum *n_f_* and the corresponding *P_t_*, that satisfy the condition are calculated by testing different *n_f_* under the minimal limit of *P_t_*. According to the standard deviation of the error, the point whether belonging to inliers is determined^25^ and as shown in Eq. (6):

(6) $$\text{point}=\left\{\begin{array}{cc} \text{inliers}, & \left|d\right|\leq t=1.96\sigma \\ \text{outliers}, & \text{otherwise} \end{array}\right.$$

when n≥2m, the error standard deviation *σ* can be estimated as follows:

(7) $$\sigma =1.4826\left(1+\frac{5}{n‐m}\right)\sqrt{med_{i}\left|d_{i}\right|}$$

where $d_{i}$ is the data error shown in Fig. 3, $med_{i}\left|d_{i}\right|$ is the median of the absolute value of error.

After pretesting, the correct model might be regarded as wrong and removed. The probability of choosing an un‐contaminated m inliers sample point is changed to ${\left(1‐\varepsilon \right)}^{m}P_{t}$, and then the Eq. (3) becomes:

(8) $$k\geq \frac{\log(1‐P)}{\log(1‐{\left(1‐\varepsilon \right)}^{m}P_{t}}$$

The impact of the pretesting process on the calculation results is transferred to the sampling process through the Eq. (8). When setting *P_t_* < 1, the corresponding samples *k* is more than that used in general RANSAC algorithm. However, the added model parameters are very few compared with the model that can be filtered out during the pretesting, since most of the model parameters were affected by outliers.

#### 2.2.3 Local optimization technique

As the criteria for stopping operation of algorithm are too idealized, a contaminated model may also get high support (more inliers) and lead to an incorrect result. The combination of local RANSAC and PCA is proposed to tackle the accuracy problem. Through pretesting technology, an initial solution and a consensus set that match can be acquired. The local RANSAC is sampling on this suboptimal inlier dataset and the new model is also tested on these data points. Unlike the ordinary RANSAC algorithms, there is no need to minimize the number of samples because sampling is done at inliers. In general, to reduce the overall computation time, the number of samples *k_L_* in local RANSAC is set between 10 and 20 times.

After using local RANSAC, an optimized model parameters and dataset of inliers can be obtained. Nevertheless, the parameters are not the best solution because it is calculated from the smallest sample subset. Therefore, the PCA is applied to obtaining the desired model fit by minimizing the error estimation of the consensus dataset. The optimized approximation of the needle shape will be performed in next experiment section.

### 2.3 Needle tip localization

**Fig. 4:**
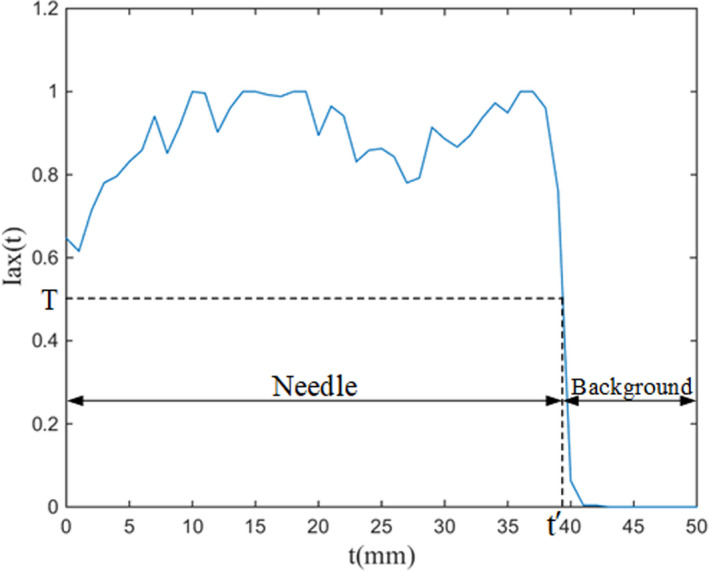
An example of needle tip localization. $I_{\mathrm{ax}}\left(t\right)$ represents the pixel intensity along the tool. A significant drop point is observed in the spectrum area corresponding to the endpoint. The threshold *T* is obtained from training data.

Once the needle axis has been identified, the intensities of all pixels along the axis are obtained and the endpoint of the needle can be found as a mutation point by the method of Barva et al.^9^ An instance of intensity profile along the estimated axis is shown in Fig. 4. Given the intensity I, the priori estimated probability distributions of the tool pixel $P(Ne\left|I)\right)$ and the background pixel $P(Bg\left|I)\right)$ are applied to determine the value T, which meet the relationship $P(Ne\left|T)\right)=P(Bg\left|T)\right)$. The estimate distribution of needle and background pixel is obtained from dataset with known tool positions.

### 2.4 Multineedles detection

**Fig. 5:**
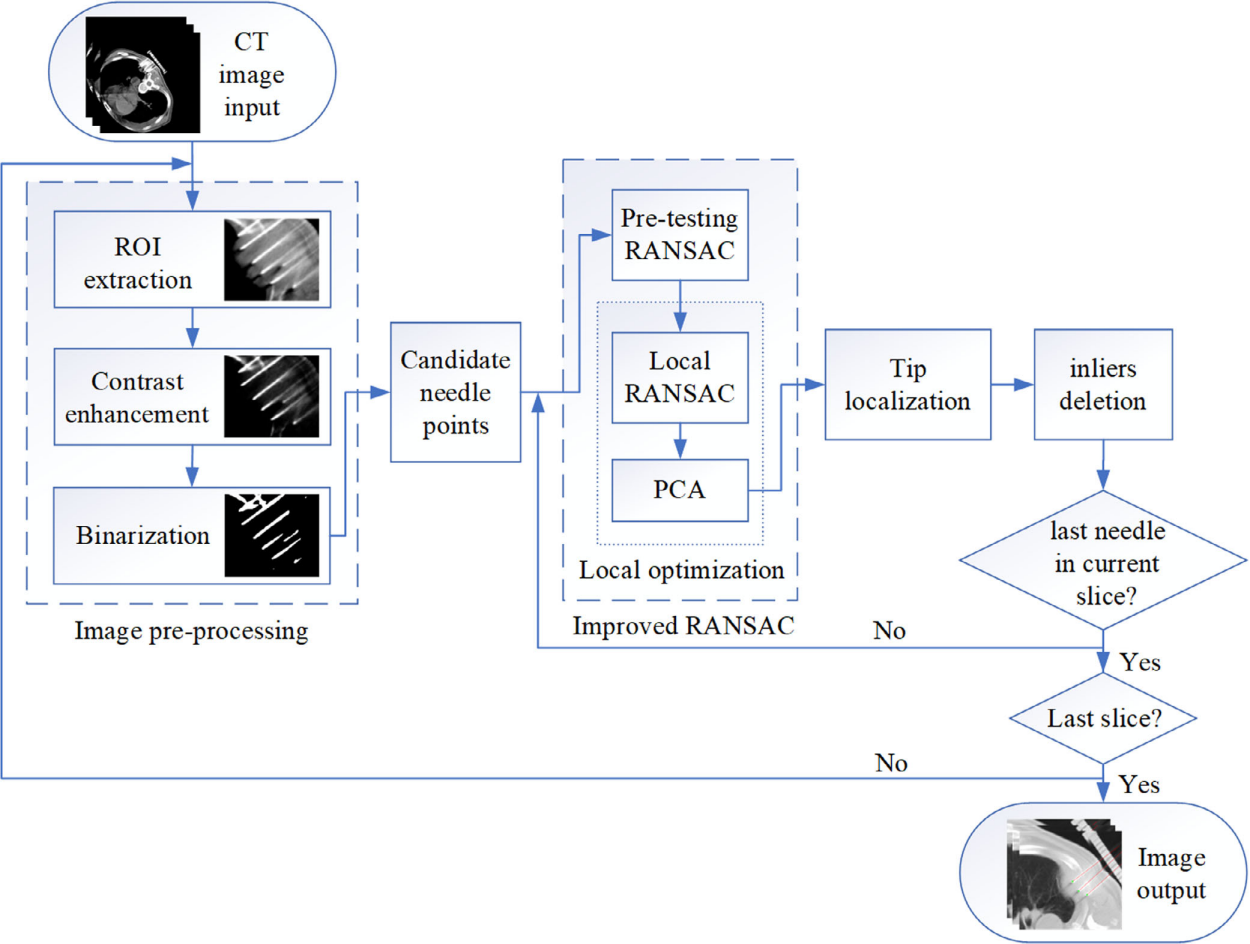
Flowchart summary of the complete needle shape estimation procedure.

Note that the RANSAC program can only take one needle at a time. To extract all the needles in a slice, we have adopted a method of successive deletion. It refers to labeling all inliers corresponding to the current optimal model after a modified RANSAC routine and removing these data from the overall sample, then using the updated sample dataset for next detection. As the sample data are updated, the outlier occupancy rate *ε* for current needle must also be changed:

(9) $$\varepsilon =\frac{N_{j}‐n_{\mathrm{en}}}{N_{j}}$$

(10) $$n_{\mathrm{en}}=\frac{N_{0}*\left(1‐\varepsilon_{\mathrm{noise}}\right)}{n_{\mathrm{needles}}}$$

where $n_{\mathrm{needles}}$ is the total needle number in current slice, $\varepsilon_{\mathrm{noise}}$ is the noise occupancy rate after image processing, $n_{\mathrm{en}}$ refers to the maximum inliers number of each needles, $N_{0}$ is the initial count of the sample dataset and $N_{j}$ is the points number after j times updating. The successive deletion method is repeated until there is no subset in the total data that satisfies the best model conditions. A flowchart showing the complete needle shape estimation steps is shown in Fig. 5.

### 2.5 Experimental methods

Three different experiments including simulation data, physical phantom images, and case images of lung interstitial brachytherapy were applied to test the performance of proposed approach. For simulation data and phantom data, the ground truths of the needle parameters were obtained through theoretical calculations. For the case data, since the actual needles information cannot be obtained, the manual segmentation results were used as the ground truths. The needle parameters obtained by the automatic detection algorithm would be compared with the ground truths to calculate the angle deviation *β* and the needle tip position deviation *ξ* to measure the performance of the algorithm.

The angle deviation *β* refers to the angle between the detected needle shaft and the actual needle shaft:

(11) $$\beta =\arccos\frac{\left|x_{s}x_{l}+y_{s}y_{l}\right|}{\sqrt{x_{s}^{2}+y_{s}^{2}}\sqrt{x_{l}^{2}+y_{l}^{2}}}$$

where $\left(x_{s},y_{s}\right)$ is the detected needle shaft vector, $\left(x_{l},y_{l}\right)$ is the actual needle shaft vector. The needle tip position deviation ξ refers to the Euclidean distance on the 2D slice between the detected needle tip position $\left(x_{2},y_{2}\right)$ and the actual needle tip position $\left(x_{1},y_{1}\right)$:

(12) $$\xi =\sqrt{{\left(x_{1}‐x_{2}\right)}^{2}+{\left(y_{1}‐y_{2}\right)}^{2}}$$

In this study, the confidence probability P was set as 99%, the sample data n for pre‐testing was chosen as 15 and the probability $P_{t}$ was set to 80%. According to empirical results, $\varepsilon_{\mathrm{noise}}$ was selected as 15%. For each experimental data, the detection algorithm was repeated 15 times for each dataset, the mean and standard deviation of related data were recorded. All algorithms were implemented in MATLAB (The MathWorks, Natick, MA) and running on an Intel(R) Core (TM) i3‐2120 3.30GHz computer.

#### 2.5.1 Evaluation on simulation data

Datasets with a size of 1500 points were generated by the computer to test our method. All the inliers came from a given straight line in each dataset. Moreover, uniformly distributed different proportions of Gaussian noise varying between 0.1 and 0.8 was added with MATLAB function “randn” in a certain area. Note that the synthetic data generated were applied only to verify the feasibility and improvement of proposed method in the data with multiplicative Gaussian noise.

#### 2.5.2 Evaluation on physical phantom images

**Fig. 6:**
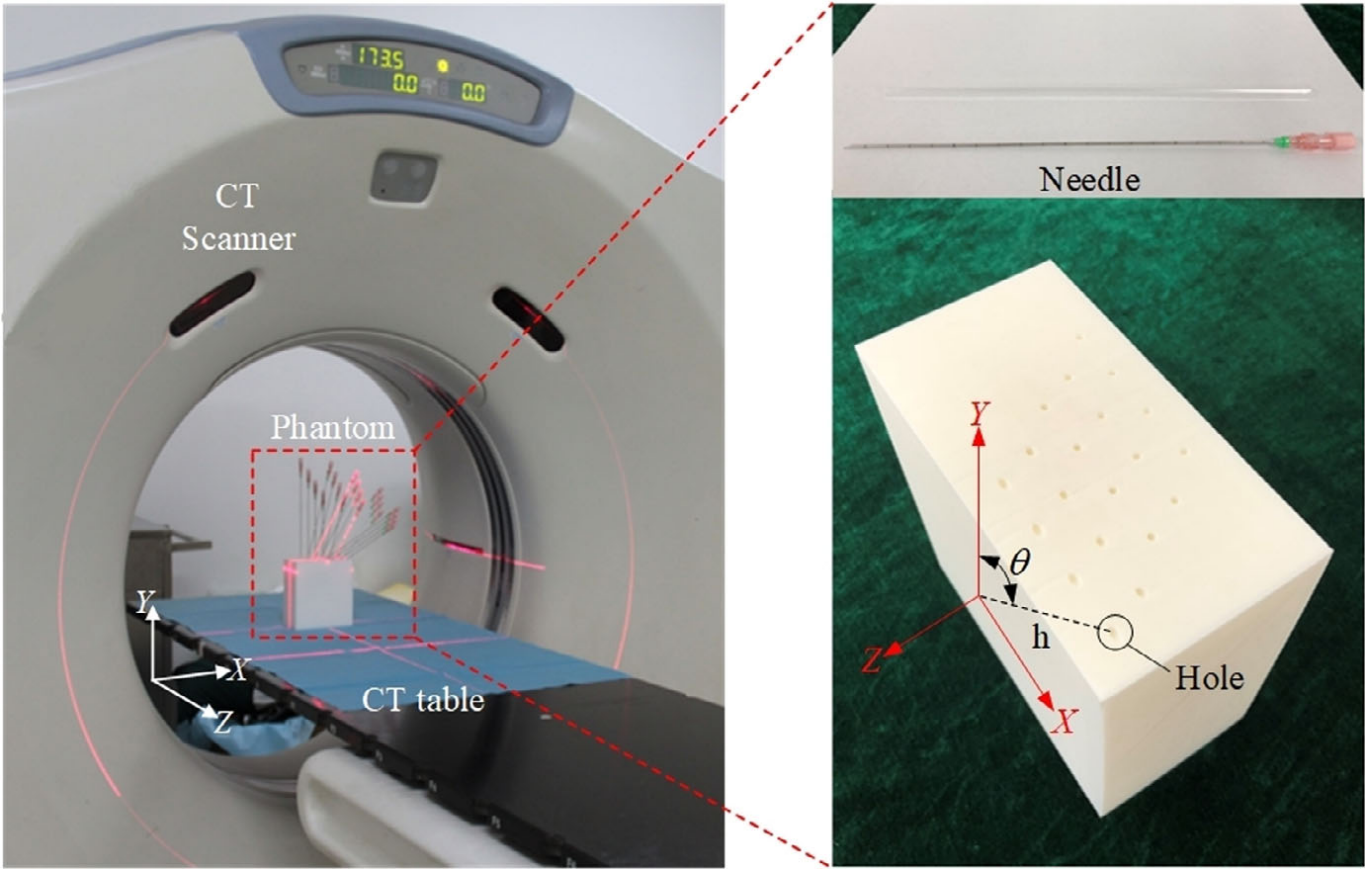
The physical phantom containing 20 needles in a CT environment. θ and h represent the insertion angle and insertion depth, respectively.

To mimic the needle insertion more closely during clinical surgery, a 3D printing technology based in‐laboratory validation phantom was devised with 20 18‐gauge brachyneedles (made in Hakko Co., Ltd, Nagano, Japan) in known orientations and endpoints. The 115 mm × 60 mm × 110 mm plate and a matrix of holes was directly formed through a digital 3D printer (made in Aurora Technology Co., Ltd, Shenzhen, China with machine error 0.05 mm). In the matrix of holes, various of insertion angle θ varying from 0° to 60° in 15° step with different insertion depth *h* = 40, 60, 80, and 100 mm were planned to test the robustness of our algorithm (as shown in Fig. 6). The validation experiment was carried out at The Second Hospital of Tianjin Medical University, 15 CT image slices of the phantom were obtained from a spiral CT system (GE LightSpeed RT) with the parameters were set to 100 kV and 150 mA. The size of the physical phantom image was 512 × 512 × *Z*, where *Z* ranged from 4 to 12 slices with a voxel resolution of 0.70 × 0.70 × 5 mm^3^.

#### 2.5.3 Evaluation on in vivo experiment

Fourteen patient CT images of lung cancer brachytherapy were also used in our experiments. All the patients were recruited from the Second Hospital of Tianjin Medical University. Moreover, details of the needle provided by medical physicist are presented in Table 1.

**Table 1:** Report of evaluation of accuracy at CT‐guided lung brachytherapy images of 14 patients.

case	Nb of needles implanted	Nb of needles picked	$\bar{h}$ (mm)	Position deviation (mm)		Angular deviation (°)		DR (%)	Segment time per needle (s)
				$\bar{\xi }$	Δξ	$\bar{\beta }$	Δβ		
1	15	14	40.679	0.670	0.202	0.940	0.657	93.33	0.215
2	10	10	76.023	0.615	0.133	1.189	0.196	100	0.293
3	14	13	68.694	0.599	0.180	1.104	0.419	92.86	0.248
4	11	10	35.621	0.757	0.218	1.324	0.390	90.91	0.225
5	14	13	85.249	0.586	0.194	0.887	0.529	92.86	0.238
6	12	12	55.723	0.708	0.119	1.156	0.488	100	0.231
7	15	14	74.861	0.594	0.216	0.911	0.518	93.33	0.206
8	12	12	64.173	0.662	0.175	1.058	0.279	100	0.241
9	13	12	48.640	0.752	0.163	1.243	0.562	92.31	0.229
10	14	13	55.872	0.809	0.107	1.257	0.518	92.86	0.234
11	11	11	63.459	0.713	0.118	1.339	0.382	100	0.217
12	12	11	59.814	0.657	0.142	0.983	0.670	91.67	0.253
13	14	13	50.351	0.699	0.174	1.167	0.348	92.86	0.244
14	16	14	75.128	0.571	0.127	0.952	0.269	87.50	0.261

$\bar{h}$ refers to the mean insertion distance of each case. $\bar{\xi }$ and Δξ represent the mean value and standard deviation in needle tip position error, respectively. $\bar{\beta }$ and Δβ represent the mean value and standard deviation in needle shaft error, respectively. DR corresponding to the detection rate.

## 3 RESULTS

### 3.1 Results on simulation data

**Fig. 7:**
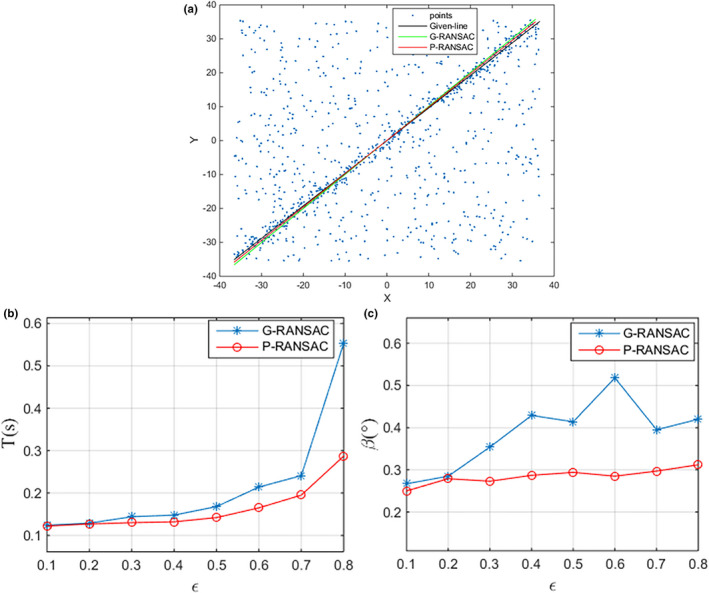
(a) The segmentation results based on P‐RANSAC and G‐RANSAC. (b) Segmentation efficiency and (c) the angular deviation *β* between the algorithm and given needle vectors from P‐RANSAC and G‐RANSAC: effect of outlier ratio *ε*.

The results of simulation data between the proposed RANSAC (P‐RANSAC) and the general RANSAC (G‐RANSAC) are shown in Fig. 7. It can be seen from Fig. 7b that the calculation time with G‐RANSAC is exponentially proportional to *ε* while slightly efficient decline occurs in P‐RANSAC with increasing error rate. When *ε* reaches 80%, the calculation consumption of P‐RANSAC is 0.287 s compared to the 0.553 s for the G‐RANSAC, which saved 48% of the segmentation time. From Fig. 7c we can find that the errors of G‐RANSAC appear to be larger at high outlier ratio and in general, decrease with decreasing outlier ratio. It is apparent that the maximum angular deviation *β* is 0.518° with *ε* = 0.6 and the minimum *β* is 0.267° with *ε* = 0.1 for G‐RANSAC. As the proposed segmentation method, the angular deviation is controlled within 0.310° and no big fluctuation happened with different error rate.

### 3.2 Results on physical phantom

**Fig. 8:**
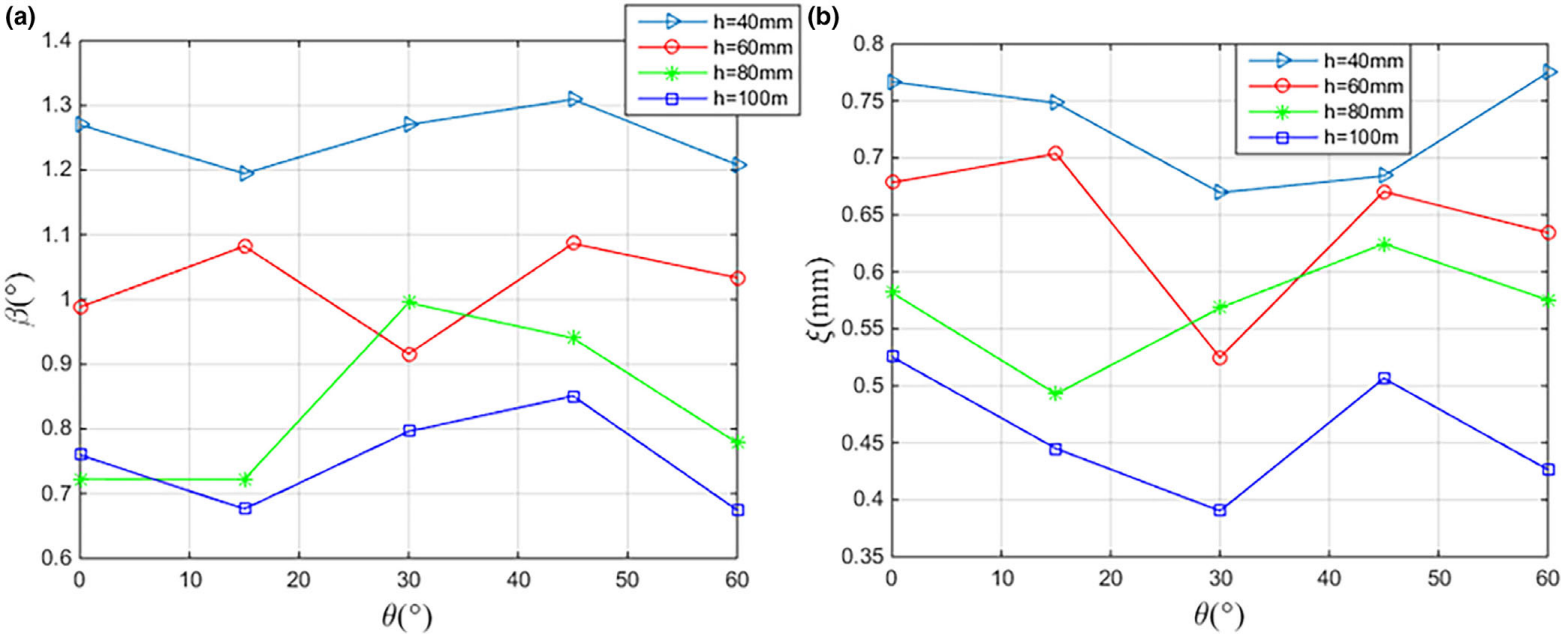
Segmentation accuracy with (a) the angular deviation *β* between the algorithm and manually segmented needle vectors and (b) the position deviation of needle tip *ξ* derived from the proposed algorithm, respectively: effect of needle insertion angulations θ and insertion distance *h*.

For each needle insertion distance *h* investigated, the observed variation of *β* and *ξ* with different insertion angulation θ is plotted in Fig. 8. The segmentation error of needle axis and needle tip is smaller at deeper insertion distances, and it increases with decreasing insertion distances. Since more available candidate points of the needle can be obtained with deeper needle depth, leading to more accurate needle shaft determination. Quantitative results also reveal that *β* and *ξ* are both insensitive to θ, and vary slightly with increasing insertion angulations. Under the different insertion angulations of the same insertion distances, the maximum variation of *β* and *ξ* is 0.273° and 0.179 mm, respectively. At the deepest insertion distance investigated (100 mm), the minimum error of automatically segmented needle shafts and endpoint positions from their counterparts of manually segmented is 0.674° (mean) and 0.390 mm (mean), respectively. At the smallest insertion distance investigated (40 mm), the errors increase to about *β* = 1.309°and *ξ* = 0.775 mm. All the needles used in physical phantom experiments are accurately identified and the mean time is 0.192 s per needle.

### 3.3 Results on in vivo experiments

**Fig. 9:**
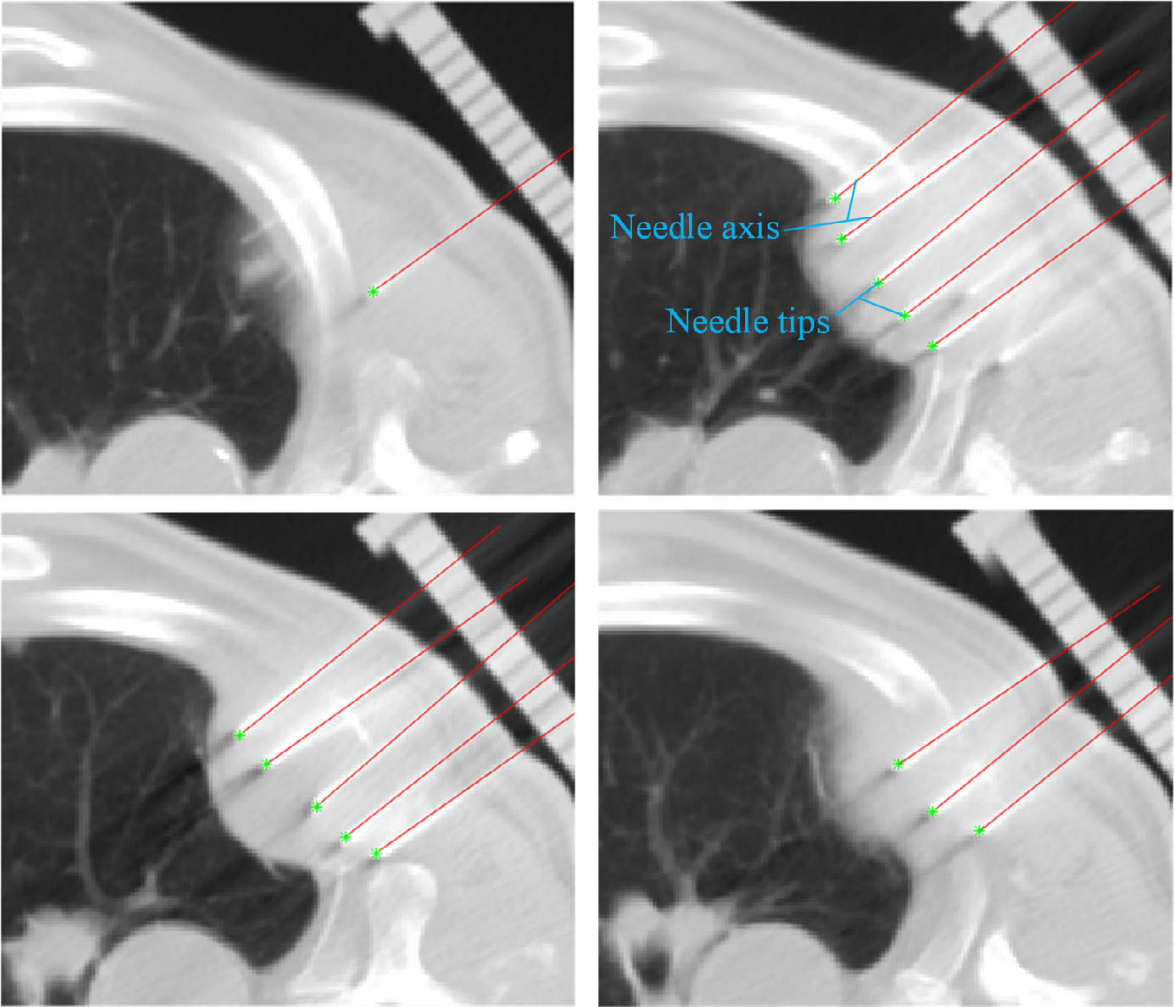
Example of needle segmentation result in a set of transverse CT images obtained during lung interstitial brachytherapy. The red line are the detected needle axes, and the green dots are the detected needle tips.

Fig. 9 illustrates an instance of the detection results for a set of CT lung brachytherapy image with our method. From Table 1, it can be found that the maximum angular deviation is 1.243 ± 0.562° and maximum tip location error is 0.757 ± 0.218 mm in all identified needles. Comparing with the previous experimental data, the error of needle configuration in patient data becomes larger. This is mainly because the mistake is not only derived from the segmentation algorithm but also from the medical physicist.

Additionally, the segmentation efficiency of the algorithm is also calculated, with an average of time consumption 0.238 s per needle. In addition, a total of 163 needles were implanted while 152 needles are successfully picked, resulting in a 93.25% picking rate. The primary factors of the failure in detecting needle are also discreetly analyzed. There are two aspects: (1) Strong interference from the background objects (ribs etc.) and needle artifact. (2) Only a small portion of needle was inserted into the tumor, which leading to limited candidate needle points can be used to needle detection algorithm. This situation often occurs in the edge of the target organ.

## 4 DISCUSSION

An automatic and efficient approach to accomplish implanted needle configuration segmentation has been described in this paper. The pretesting, local optimization, and successive deletion technique are the main idea applied in modified RANSAC routine. The experimental results demonstrate that proposed algorithm is feasible in simulation data with a series of points, in physical phantom with a uniform background and in patient’s slices with complex as well as nonuniform background noise.

In simulation results, preview model parameters evaluation technology speeds up the segmentation process by prescreening the model with fewer points and the acceleration process is especially noticeable when the percentage of outlier is high. This shortening of time is noticeable for picking up multiple needles in one time. Because the other needle inliers are also belong to noise for current model in the first few needles extraction. In other words, the outlier ratio is higher in algorithm early period.

In our physical phantom experiment, the influence of different insertion angles and insertion distances on the performance of detection method are analyzed. It can be seen that the difference in accuracy of needle axis and endpoint segmentation is mainly caused by target amount of information, which depends on the insertion depth. Despite aforementioned factor, the algorithm we proposed still has higher accuracy and better robustness, since the error in determining the needle tip is less than 0.78 mm and the needle orientation is less than 1.35°. Comparing with previous related study in needle orientation, the experimental result illustrates more competitive than 2.3° (Cool’s method).^26^ Moreover, for the error of needle tip, the evaluation value is smaller than 0.8 mm (Ding’s algorithm)^16^ and 1.43 mm (Qiu’s algorithm).^27^

For the actual brachytherapy images, the needle tip location and orientation error are controlled within 1 mm and 2° for most cases. The submillimeter accuracy is sufficient for intended brachytherapy clinical practice. Meanwhile, the whole failure rate is 6.75%, which is completely reasonable for a random iterative algorithm (RANSAC), and this value is more competitive than 33% (Uhercík’s result)^19^ and 16% (Qiu’s result).^27^ As for the segmentation efficient, the average processing cost by our approach is 0.238 s per needle. It is much shorter than other algorithms, the mean time in reference 11 is 7.3 s, 26 is 3‐5 s, and 19 is 0.64 s. This time consumption is perfectly acceptable compared to a surgery with few hours. In addition, based on our knowledge, the application of needle detection based on CT images for brachytherapy has not yet been reported.

It is important for the best therapeutic effect in lung brachytherapy to realize intraoperative dose replanning before seeds implanted into pathological tissue. In this paper, a CT image‐based quick and accurate needle reconstruction technology has been described, which provides a new idea for dose correction. Once identified all needles, the original plan (all the seeds) can be imported based on the correspondence between preoperative and intraoperative needle, and then ameliorate dose distribution by adjusting primary seeds position as well as adding extra needles and seeds. On the other hand, an image measurement tool in auto‐determining the needle tip position and needle orientation is available for medical physicist. If obvious positioning mistakes are detected, the remedy like withdraw or reinsert the needle will be considered. In addition, the proposed method provides the flexibility to segment other parametric objects through redefining the model function and corresponding error term.

Although our algorithm can detect multiple needles precisely at once, there are still some failures. Thus, to guarantee a robust segmentation, an advanced initialization (preimage processing) is desired in our application. It is important to note that the needle deformation in Z direction was not considered in this article. There are a few reasons for current lung brachytherapy: from one perspective, the needle insertion depth will be decreased as much as possible by adjusting the patient position in surgery to increase the control of needle. From another perspective, changing the bevel orientation of the needle tip during needle insertion contributes to diminishing the deformation problem. Through these intraoperative methods, the deviation of needle position in Z direction is much less than the deviation in X and Y direction. In our future work, the possibility of combining with the coronal or sagittal slice will be considered to further tackle this problem and achieve a 3D parametric representation of the needle shape.

## 5 CONCLUSION

An improved RANSAC algorithm has been presented in this paper for CT image‐based needle segmentation. Preview model parameters evaluation, local optimization combining local RANSAC and PCA, and successive inlier deletion are the key technologies applied in this method, which were tested in simulation data, physical phantom, and brachytherapy case. Meanwhile, the promising experimental results with short calculation consumption and higher accuracy meet the requirement of clinical treatment. We expect that, with the image‐based automatic needle placement, a more reliable dose optimization module will be integrated into the brachytherapy treatment planning system.

## AUTHOR CONTRIBUTIONS

Yongnan Zheng made the conception and design of the study, drafted the article, and revised it critically for important intellectual content. Shan Jiang and Zhiyong Yang made the approval of the version. Lin Wei analyzed and interpreted the data.

## CONFLICT OF INTEREST

The authors declare that they have no conflict of interest.

## ETHICAL APPROVAL

All procedures performed in studies involving human participants were in accordance with the ethical standards of the institutional and/or national research committee and with the 1964 Helsinki declaration and its later amendments or comparable ethical standards.

## 6 INFORMED CONSENT

Informed consent was obtained from all individual participants included in the study.
